# Shield construction safety risks and their interrelations analysis of subway tunnel undercrossing a river based on Grey-DEMATEL-ISM

**DOI:** 10.3389/fpubh.2025.1536706

**Published:** 2025-04-10

**Authors:** Yu Xue, Xun Luo, Hujun Li, Jie Liu

**Affiliations:** ^1^Shaanxi Provincial Department of Ecology and Environment, Xi’an, Shaanxi, China; ^2^School of Medicine, Henan Polytechnic University, Jiaozuo, Henan, China; ^3^School of Civil Engineering, Henan Polytechnic University, Jiaozuo, Henan, China

**Keywords:** subway tunnel shield construction, safety risks assessment, undercrossing a river, safety risks list, interrelations structure of safety risks, grey number, DEMATEL-ISM

## Abstract

A large number of subway projects were designed and constructed by China’s city government and more subway tunnels can hardly avoid undercrossing a river. Shield construction of subway tunnel undercrossing a river (STUR) is often in a complicated geological environment, which cause the construction process more prone to safety accidents. Hence, it is necessary to investigate the shield construction safety risks (SCSRs) of STUR in advance. The paper identified the related SCSRs of STUR using literature review and experts’ discussion, put forward a hybrid approach to examine the safety risks and their interrelations by integrating grey number, DEMATEL and ISM, and then selected a case to validate the feasibility of the proposed safety risk list and hybrid approach. Research results show that (a) a SCSRs list of STUR was identified, including 4 safety risk categories and 32 safety risks; (b) the proposed safety risk list and hybrid approach can be utilized to identify safety risks and analyze the significance and interrelation structure of the identified SCSRs of STUR; (c) most natural environment safety risks (levee, quick sand layer and high water pressure), management environment safety risks (safety institution and safety organization & duty) and manager safety risks (safety inspection, safety management competency) are salient safety risks; (d) the established safety accident causation structure, from the bottom up, includes environment safety risk level, personnel safety risk level, technology safety risk level, machine safety risk level, and accident level. The research can enrich the theoretic knowledge in shield construction safety risk analysis of STUR, and offer references for safety managers to carry out onsite scientific and effective safety management of STUR shield construction.

## 1 Introduction

Recently, the Chinese government has substantially expanded its investment in urban infrastructure to address the evolving needs of its population (1–3). As a cost-effective and sustainable mode of urban transportation, subways have been rapidly developed nationwide, characterized by their safety, reliability, and energy efficiency (4, 5). However, due to the complexity of urban environments, which often necessitates river crossings for subway routes, tunneling under rivers remains a technically challenging task. Despite the predominant employment of shield machines in tunnel construction, the construction phase of river-crossing tunnels presents unique safety risks due to complicated geological and hydrological conditions. Such risks often manifest as catastrophic incidents, including tunnel collapses and water inrushes (6–8), with severe consequences such as loss of life, injuries, and substantial property damage (9, 10). A notable case was the Foshan Metro Line 2 accident in 2020, where a water inrush resulted in 12 fatalities, 8 injuries, and extensive infrastructure damage, highlighting the critical need for systematic risk assessment. Therefore, this study aims to investigate the shield construction safety risks (SCSRs) associated with subway tunnel undercrossing rivers (STUR) to establish preventive measures and mitigation strategies.

Numerous studies have investigated the safety risks associated with subway shield construction, encompassing both generic scenarios and specific contexts such as complex overburden stratigraphy (4, 11, 12), proximity to existing structures (13–16), adjacent tunneling (16, 17), pipeline crossings (18), and bridge underpasses (19). The extant research primarily concentrates on three core dimensions: risk identification, quantitative risk assessment, and inter-risk correlation analysis. Dominant frameworks for risk characterization include the “human-machine-environment” triad and its derivatives, while evaluation methodologies frequently employ multidisciplinary approaches such as the Analytic Hierarchy Process (AHP) (20), composite weighting algorithms (19), fuzzy synthetic evaluation (21, 22), matter-element modeling (13, 23), cloud theory (20), Bayesian Networks (BN) (24, 25), and Back Propagation Neural Networks (BPNN) (26–28). Although some investigations have probed inter-risk relationships through systematic hierarchy techniques (ISM and ISM-DEMATEL) (29–32) and network-based paradigms (BN, Fuzzy BN, event factor networks) (33–35), scholarly attention to STUR-specific safety challenges remains conspicuously scarce (28, 36). Besides, the identified risks in these studies are often incomplete and fragmented, and they tend to overlook the safety impacts from the perspectives of personnel and methodology (technology). Furthermore, subway shield construction accidents are typically the result of the interaction between multiple safety risks, yet few studies have delved into the interrelations among these risks in the context of STUR. Consequently, there is a need for further investigation into the safety risks and their interconnections in STUR, which would enable project management teams to better anticipate and control potential safety hazards.

This study focuses on SCSRs of STUR, aims to address the two aforementioned critical gaps in river-crossing shield tunnel risk assessment: (1) the lack of comprehensive safety risks list for shield construction of STUR; (2) the limitation of traditional approach (e.g., qualitative risk matrices or single-theory quantitative models) in capturing multi-dimensional interactions between geological, hydrological, and operational risks. Initially, we identify these safety risks by employing a “personnel-machine-technology-environment” framework, leveraging both a comprehensive literature review and expert consultations. Subsequently, we introduce a hybrid analytical approach that integrates grey number theory, Decision-Making Trial and Evaluation Laboratory (DEMATEL), and Interpretive Structural Modeling (ISM) to analyze the identified risks and their interrelationships. This method is used to develop a causation structure model that elucidates the potential pathways leading to safety accidents. To validate the effectiveness and applicability of our proposed approach, we apply it to a case study of the Guangzhou Metro Line 18, which crosses under the Pearl River.

The superiority of the hybrid approach is that it provides integrated frameworks that combine uncertainty analysis (grey number theory), causal relationship mapping (DEMATEL), and hierarchical decomposition (ISM) to prioritize risks systematically. Grey Number Theory handles uncertainties well when data is incomplete and relatively less, unlike Bayesian Networks that require extensive data; DEMATEL helps analyze interdependencies among risks more effectively than Fuzzy AHP by revealing how different risks influence each other. ISM provides a clear hierarchical structure of causal relationships, making it easier to interpret compared to complex Bayesian Networks. Together, these methods offer a robust and practical solution for risk assessment in STUR.

The findings of this research will provide valuable insights for safety management personnel and policy-makers in subway shield construction, enabling them to better understand the underlying mechanisms of safety incidents. This understanding can help in the policy making and the proactive establishment of safety intervention measures, ultimately contributing to the prevention or reduction of safety accidents during subway tunnel construction.

## 2 Literature review

### 2.1 Safety risk identification during subway tunnel shield construction

Safety risk identification is a fundamental step in the safety risk assessment process. Previous research has explored the safety risks associated with subway tunnel shield construction from multiple angles (15, 18). These studies suggest that the external environment, shield construction technology, and on-site management are key factors contributing to safety risks in shield tunnel projects. An “equipment-environment-management” identification framework has often been employed (11, 12, 16, 19). For example, Hu et al. (11) analyzed safety risks during subway shield construction beneath soft overburden, identifying geological complexity, groundwater conditions, minimum overburden thickness, minimum radius of curvature, construction speed, proximity to surrounding structures, and the level of construction management as critical risk factors. Similarly, Zhai et al. (19) investigated safety risks when constructing a subway shield tunnel near an existing bridge, highlighting the importance of geographical and hydrological conditions, shield construction parameters, tunnel and bridge conditions, as well as organizational and management-related risks. The complexity of subway shield construction has led scholars to emphasize the significance of the “personnel-equipment-environment” system in addressing these challenges. Liu et al. (4) and Chen et al. (5) focused on safety risks during the construction of subway shields under complex overburden layers, employing the “personnel-equipment-environment” framework for their analysis. An extended version of this framework, the “personnel-equipment-environment-management” model, was utilized by Wu et al. (37) and Pan et al. (38) to further investigate safety risks in shield construction. Furthermore, a more comprehensive approach, the “personnel-equipment-material-technology-environment” framework, has been adopted by Li et al. (21) and Fan and Wang (23) to systematically identify and assess safety risks in subway shield construction projects. This systematic framework allows for a more holistic understanding of the interplay between various elements, thereby enhancing the accuracy and effectiveness of safety risk assessments.

### 2.2 Safety risk evaluation during subway tunnel shield construction

Safety risk assessment primarily involves the development of models that clearly outline the process for calculating risk. Given the multitude of safety risks within the index framework, two critical aspects in model design are the determination of weights and the methods for measuring safety risks. Initially, the Analytic Hierarchy Process (AHP) was a popular choice for establishing weight systems. For instance, Li et al. (21) utilized AHP to determine the relative importance of various safety risks in slurry balancing shield construction. Over time, there has been a shift towards more objective approaches to reduce subjectivity in weight assignment. Fan and Wang (23), for example, incorporated the ISM-DEMATEL and Shapley value methods to account for interdependencies among different safety risks. Zhai et al. (19) combined G1 and CRITIC methods to create a composite weighting system for assessing safety risks when constructing near an existing bridge. Regarding the measurement of safety risks, several quantitative techniques have been identified. The fuzzy comprehensive evaluation method is frequently used for assessing safety risks in shield construction (21, 22). Ren et al. (22) applied this approach to evaluate overall safety risks during construction near an existing structure. Another widely adopted technique is the matter-element method (13, 23), which is effective for linking specific risks with their corresponding criteria (3). More recently, Bayesian networks (16, 39) and cloud models (12, 37) have been introduced to address uncertainties in risk assessment. Wu et al. (16) integrated fuzzy Bayesian analysis with evidence theory to assess risks in subway shield construction beneath an existing tunnel. Meanwhile, Wu et al. (37) and Chen et al. (12) employed cloud models and extended cloud theory, respectively, to analyze safety risks in projects adjacent to existing buildings. Additionally, Monte Carlo simulations (19) and System Dynamics (SD) (38) have been applied to simulate the probabilistic sampling processes and dynamic interactions between different safety risks, further enhancing the accuracy and reliability of risk assessments in shield construction.

### 2.3 Interrelation analysis of safety risks in the construction area

The simple measurement of construction safety risks using risk values alone appears insufficient because this approach neglects the interactions and transmission among safety risks (40, 41). Consequently, recent efforts by scholars have focused on analyzing the interrelations among these risks from a systemic perspective (29, 42). These studies aim to identify significant safety risks and investigate the formation of related safety accidents through a comprehensive framework. Some researchers have adopted a hierarchical system view, essentially following Heinrich’s safety accident causation theory (43). They identified the construction safety risks involved, analyzed the system hierarchy of these risks, and presented a multi-tier causation model for associated safety accidents. System engineering-type approaches, such as ISM, DEMATEL, and Fuzzy ISM, were frequently employed to construct these multi-tier hierarchies (29–32). For instance, Shi et al. (31) examined the safety risks of a coal mine construction project by integrating ISM and DEMATEL methods, establishing a six-tier hierarchy model to analyze safety transmission paths. Other researchers have opted for a network view, emphasizing that safety risk networks are an appropriate mathematical tool for illustrating the complex interrelations among safety risks (40, 44, 45). Once these safety risk networks are established, researchers can infer the causal chain between a specific safety risk and a type of safety accident. Previous studies have primarily used Bayesian Networks (BN) and complex networks, including Fuzzy BN and BN-Human Factors Analysis and Classification System (BN-HFACS), to analyze these safety risk networks (33–35). For example, Xu et al. (40) developed a factor-event network for subway construction, identifying key safety protocols and safety transfer processes within the safety network.

Comparing the two approaches mentioned above, although network-based studies may illustrate a greater number of connections among safety risks, models based on the system hierarchy view tend to be more concise and practical. Network-based models can sometimes highlight insignificant connections, thereby increasing model complexity and hindering practical application. In contrast, system hierarchy-type models aim to categorize safety risks and establish causal relationships between these categories in a structured, progressive manner. Consequently, this research integrates grey number theory into ISM-DEMATEL to analyze the interrelations among identified shield construction safety risks during subway river-crossing (SUR) projects.

## 3 Shield construction safety risk identification

### 3.1 The framework for safety risk identification

**Figure 1:**
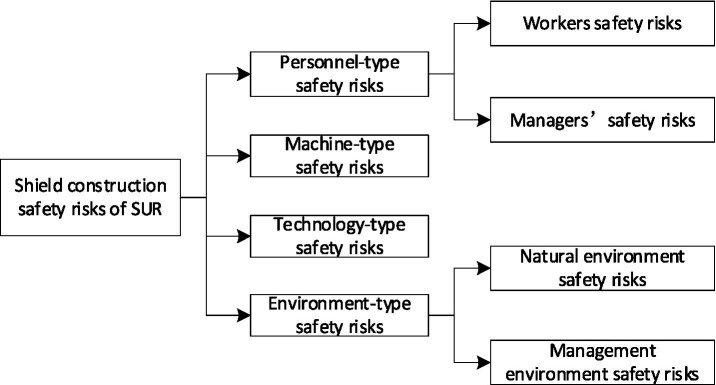
Identification framework of SCSRs of STUR.

Although there are few studies investigating the SCSRs of STUR, existing literature can shed light on the identification ideas in this research. Previous literature has identified the framework of “personnel-machine-material-technology-environment” as a relatively systematic framework (21, 22). After consulting onsite experts, material-related safety risks should not be considered because after several material inspections, non-standard materials should not be used. Thus, in this study, we selected the framework of “personnel-machine-technology-environment” to identify the related SCSRs of STUR (refer to Figure 1).

### 3.2 The process for safety risk identification

Based on the established safety risk identification framework, this research adopted a two-step approach to identify the SCSRs of STUR. Initially, we performed a comprehensive literature review to compile a list of relevant safety risks. Following this, we convened an expert panel to evaluate and refine the pre-identified risks, ensuring a thorough and accurate assessment.

#### 3.2.1 Step 1: safety risk identification based on literature review

CNKI and Scopus were selected as retrieval databases, and the search strategy is (“safety risks” AND “shield construction”) OR (“shield construction” AND “river*”). The initial search shows 86 English papers and 65 Chinese papers. After a systematic review of the titles, abstracts, and texts, 57 documents were retained, consisting of 34 English papers and 23 Chinese papers. Safety risks were initially gathered from the retained documents.

#### 3.2.2 Step 2: safety risk evaluation and enrichment based on expert discussions

To ensure comprehensive and authoritative identification and evaluation of shield safety risks associated with Subway Tunneling Under Rivers (STUR), we organized a panel of 15 safety management experts from universities, enterprises, and consulting firms. The expert group was carefully selected to include professionals from diverse backgrounds: three professor-level academics with extensive research experience and publications in urban underground engineering, ensuring a solid theoretical foundation; five senior engineers with over 10 years of practical project management experience in shield construction, providing real-world operational insights; and seven site engineers well-versed in current safety regulations and technical standards, who have hands-on experience supervising multiple metro projects, thus guaranteeing compliance with legal norms and best practices.

Each expert’s professional expertise was validated through a rigorous selection process. This included reviewing their resumes and research contributions, conducting initial interviews to assess their understanding of shield construction and risk management, and examining their past project experiences and performance records, particularly in complex geological conditions. By combining robust theoretical knowledge with practical experience, this panel ensured the validity and reliability of the identified safety risk list. As a result, we identified a Shield Construction Safety Risk (SCSR) list for STUR projects, which is summarized in Supplementary Table 1.

## 4 Shield construction safety risks analysis approach

We designed an approach to analyze the safety risks and their interrelations by integrating grey theory, DEMATEL and ISM. The grey theory was used to establish the direct impact matrix, the DEMATEL was applied to evaluation the significance of the safety risks, and the ISM was used to examine the causality associations of the safety risks. The processes of the approach are as below.

### 4.1 Determining the direct impact matrix base on grey theory

The proposed approach follows the below steps to gain the direct impact matrix.

#### 4.1.1 Establishing the grey association matrix

We firstly use *F* to denote the shield construction safety risk set, and $f_{i}\in F\;\left(i=1,2,\cdots ,n\right)$ denote each single safety risk. M experts are invited to evaluate the impact of $f_{i}$ on $f_{j}$ based on the 5-degree evaluation rule, and the impact can be expressed by $e_{ij}^{k}$ . The 5-degree grey numbers are introduced to represent the evaluation results. The relationships between the grey numbers and the evaluation degree are presented in Supplementary Table 2.

#### 4.1.2 Standardizing the grey association matrix

The standardized upper bounds and lower bounds of the grey association matrix can be calculated using Equations 1, 2 in Supplementary material (46, 47).

Where $\underline{⊕}e_{ij}^{k}$ and $\bar{⊕}e_{ij}^{k}$ denote, respectively, the upper bound and lower bound of the grey number $e_{ij}^{k}$ ; $\min\underline{⊕}e_{ij}^{k}$ and $\min\bar{⊕}e_{ij}^{k}$ denote, respectively, the minimum values of $\underline{⊕}e_{ij}^{k}$ and $\bar{⊕}e_{ij}^{k}$ ; $\max\bar{⊕}e_{ij}^{k}$ denotes the maximum value of $\bar{⊕}e_{ij}^{k}$ ; $\underline{⊕}{\tilde{e}}_{ij}^{k}$ and $\bar{⊕}{\tilde{e}}_{ij}^{k}$ denote, respectively, the standardized $\underline{⊕}e_{ij}^{k}$ and $\bar{⊕}e_{ij}^{k}$ .

#### 4.1.3 Calculating the clear values and clear association matrix

The clear values of the grey association matrix and the clear association matrix can be gained by using Equations 3, 4 in Supplementary material (46, 47).

Where $y_{ij}^{k}$ denotes the clear value of the grey association matrix; and ${\bar{e}}_{ij}^{k}$ denotes the value of the clear association matrix.

#### 4.1.4 Determining the direct impact matrix

The direct impact matrix can be obtained by calculating the mean of the *K* clear association matrices. Let $e_{ij}^{\prime }=\frac{1}{K}\underset{k=1}{\sum }{\bar{e}}_{ij}^{k}$ , the direct impact matrix $E^{\prime }$ can be expressed using Equation 5 in Supplementary material.

Where $e_{ij}^{\prime }$ denotes the direct impact of $f_{i}$ on $f_{j}$ .

### 4.2 Analyzing the shield construction safety risks base on DEMATEL

#### 4.2.1 Calculating the normalized impact matrix B

The maximum normalization method was used to normalize the matrix $E^{\prime }$ . The calculating method was presented in Equation 6 in Supplementary material.

#### 4.2.2 Calculating the comprehensive impact matrix

The comprehensive impact matrix can be calculated using Equation 7 in Supplementary material.

Where $t_{ij}$ represents each element in the comprehensive matrix T , I is the identity matrix, and ${\left(I-B\right)}^{-1}$ is the inverse matrix of I−B .

#### 4.2.3 Calculating the center degree and cause degree of the safety factors

The center degree $CD_{i}$ and cause degree $RD_{i}$ of the safety risks can be calculated by using Equations 8–11 in Supplementary material.

### 4.3 Analyzing the interrelation structure of shield construction safety risks base on ISM

#### 4.3.1 Calculating the overall impact matrix H and the accessibility matrix R

The overall impact matrix H reflects the overall impact relationship of various safety risks in the risk framework, and the calculation process was presented in Equation 12 in Supplementary material.

Where I is the identity matrix and T is the comprehensive impact matrix.

Based on the overall matrix H , the accessibility matrix R can be gained by using Equation 13 in Supplementary material.

In Equation 12 in Supplementary material, λ is a threshold index. The value of λ is generally determined by experts according to the actual situations.

#### 4.3.2 Determining the hierarchical structure of the safety risks and establish the causation model of safety accidents

According to the reachable matrix *R*, the reachable set and antecedent set of each safety risk can be obtained, denoted as *R*(*Si*) and *Q* (*Si*). Thus, the common set of each safety risk can be calculated. Then the interrelation structure of the safety risks can be gained by sequentially deleting the safety risk whose common set is empty class.

## 5 Case study

### 5.1 Project overview

The subway interval between Pazhou West Station and Xiancun Station on Guangzhou Metro Line 18 spans approximately 3,000 meters. The tunnel roof is buried at a depth of 20 to 30 meters. The outer diameter of the tunnel lining segments is 8.5 meters, with an inner diameter of 7.7 meters, segment thickness of 0.4 meters, and segment width of 1.6 meters. Notably, this section includes a traverse through the Pearl River’s frontal channel over a distance of about 1,600 meters, following an S-shaped curve. The river surface width ranges from 350 to 550 meters, with water depths varying between 4 and 8 meters. This area constitutes part of the alluvial plain of the Pearl River Delta. The shield tunnel passes through strata primarily composed of moderately weathered argillaceous siltstone and slightly weathered conglomerate. The minimum turning radius of the alignment is 600 meters, with a maximum longitudinal gradient of 15‰. The vertical clearance between the tunnel roof and the riverbed ranges from 18 to 28 meters.

### 5.2 Identify and assess the safety risks of the project based on experts’ evaluation

The subway section crossing under the Pearl River is considered a high-risk segment of the line between Pazhou West Station and Xiancun Station. Prior to this river crossing, the project management team convened a panel of 15 experts to identify and evaluate the safety risks associated with this critical phase. The experts were tasked with assessing both the likelihood and potential severity of the identified risks.

Following an on-site inspection, the experts reviewed the project documentation and discussed specific project issues with the project managers. Each expert was then provided with a detailed safety risk checklist, which included all pre-identified risks listed in Supplementary Table 1. Using a 5-point Likert scale (where 1 indicates low risk and 5 indicates high risk), the experts independently identified and rated 30 potential risks. Cohen’s Kappa coefficients (*κ*) were calculated for each risk item, with κ ≥ 0.7 indicating substantial agreement among the experts. After conducting this statistical analysis, 21 safety risks were identified and are presented in Supplementary Table 3.

### 5.3 Analyzing the shield construction safety risks of the project

**Figure 2:**
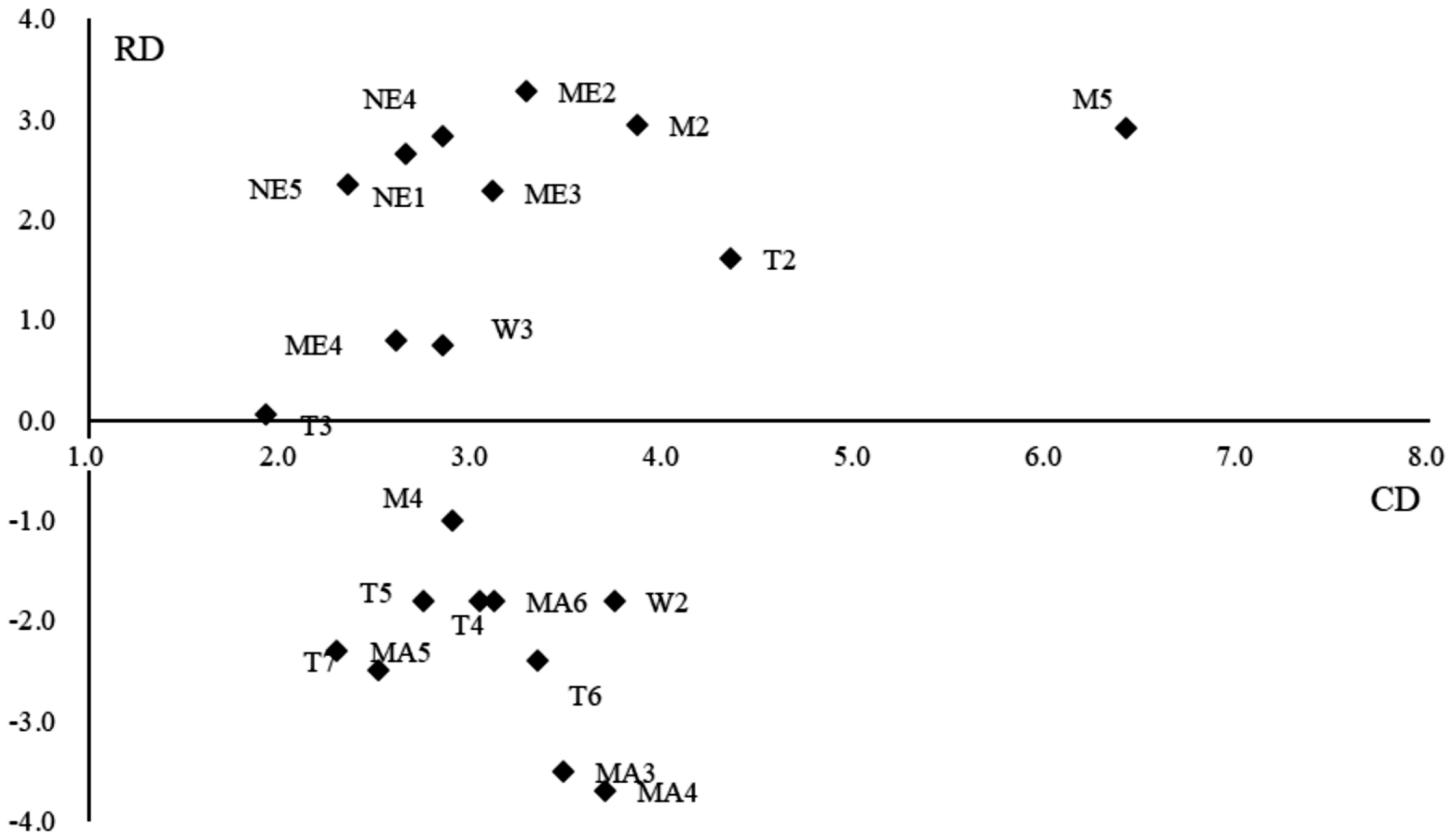
The CD-RD diagram.

The 15 experts also were invited to assess the mutual impacts among the identified safety risks and each expert’ assessment results were transformed into grey number based on transforming rule listed in Supplementary Table 2. The direct impact matrix can be calculated by following the Equations 1–5 in Supplementary material and the direct impact matrix was presented in Supplementary Table 4. Besides, by using the Equations 6, 7 in Supplementary material, we computed the comprehensive impact matrix (see in Supplementary Table 5). Thus, the center degree and cause degree of each safety risk were calculated based on the Equations 8–11 in Supplementary Table 5 and was presented in Supplementary Table 6. Then, the center degree-cause degree graph can be drawn and presented in Figure 2.

The CD-RD diagram illustrates the net cause and effect relationships of safety risks. As depicted in Figure 2, the majority of managerial safety risks, natural environment safety risks, and management environment safety risks fall into the net cause group, suggesting that these risks have a significant impact on other safety risks. Conversely, most technology-related and machine-related safety risks are classified under the net effect group, indicating that these risks are frequently influenced by others.

In the net cause group, “safety inspection” (M5), “incomplete geological and hydrological investigation” (T2), and “safety management competency” (M2) exhibit higher CD values. During on-site safety management processes, safety inspections enable managers to identify potential hazards related to workers, machinery, technology, and the environment. Therefore, safety inspections are crucial for identifying risks affecting worker safety, machine-related safety, and technology-related safety, as shown in Supplementary Table 3. A thorough geological and hydrological investigation provides detailed insights into complex geological and hydrological conditions, furnishing clients with comprehensive information about underground structures (such as pipelines, voids, and large rocks). This is essential for developing project design plans and determining construction technologies and safety measures. Thus, “incomplete geological and hydrological investigation” is an important risk influencing many machine-type and technology-type safety risks (refer to Supplementary Table 5). “Safety management competency” refers to the knowledge and skills of management personnel in construction safety management. Improved competency enables managers to effectively oversee unsafe behaviors and efficiently address safety issues related to machinery and technology during preparation and construction phases. Therefore, “safety management competency” was identified as a key safety risk and an antecedent factor causing many worker, machine, and technology-related safety risks (refer to Supplementary Table 6).

Additionally, besides “safety management competency” (M2) and “safety inspection” (M5), “levee” (NE1), “quicksand layer” (NE4), “high water pressure” (NE5), “safety institution” (ME2), and “safety organization and duty” (ME3) have higher RD values. These indicate that they are antecedent factors causing many other safety risks. “Safety institution” and “safety organization & duty” are institutional and organizational prerequisites for safety management during construction, providing the foundation for employing safety-related staff and organizing safety management tasks. Hence, these are fundamental safety risks within the system. “Levee,” “quicksand layer,” and “high water pressure” describe potential unsafe influences from the natural environment. Shield construction undercrossing rivers often faces poor soil layers and high water pressure. Levees are critical waterproofing and structural support elements; improper protection can lead to cracks and uneven settlement of foundations. Management personnel should enhance safety communication, inspections, and develop specific construction plans to prevent improper shielding. Thus, “levee” may cause potential managerial, machine-related, and technology-related safety risks (refer to Supplementary Table 3).

The CD-RD diagram (see Figure 2) categorizes and ranks safety risks based on their centrality and causal influence. Practitioners can utilize this diagram to facilitate their own safety risk management. Firstly, it helps prioritize high-impact risks. Safety risks in the upper-right quadrant require immediate intervention due to their systemic influence. For example, if “High Water Pressure” is identified, engineers might allocate budgets to install real-time piezometers along the tunnel alignment. Secondly, it aids in optimizing resource allocation. According to the CD-RD diagram, safety risks with lower CD and RD values (e.g., “ventilation system not working”) can be delegated to secondary teams, freeing resources for critical tasks. Lastly, the CD-RD diagram can be used for monitoring risk evolution. Updating the diagram with new data during construction allows for dynamic adjustment of mitigation strategies.

### 5.4 Analyzing the interrelation structure of the identified safety risks of the project

**Figure 3:**
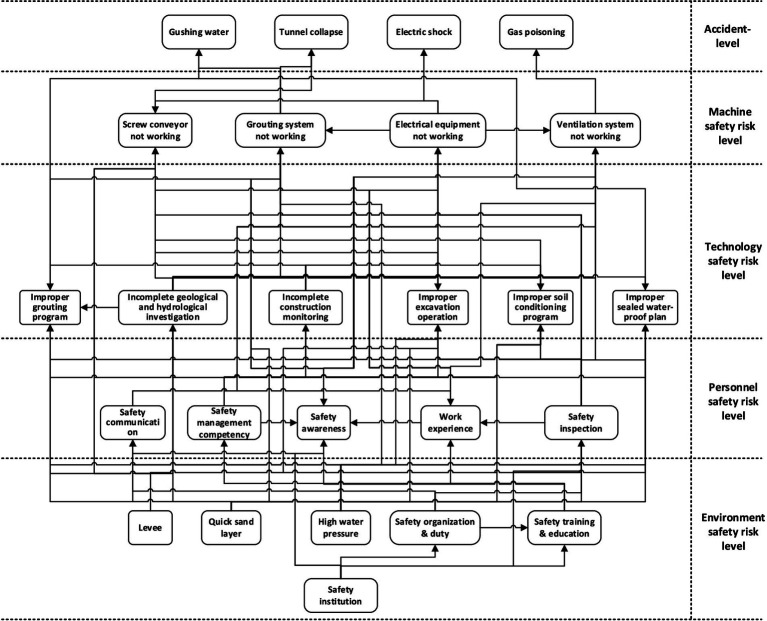
The potential safety accident causation structure of the project.

In this sector, we applied the ISM to analyze the interrelation structure of the identified safety risks and thus established the potential safety accident causation structure of the project. Firstly, the overall impact matrix was calculated based on Equation 12 in Supplementary material. Second, we selected the average of the values in Supplementary Table 3 as *λ* and used Equation 13 in Supplementary material to compute the accessibility matrix, which is presented in Supplementary Table 7. Using MATLAB to implement the process explained in Step (9, Determining the hierarchical structure of the safety risks and establish the causation model of safety accidents) and connecting the direct safety risks to related safety accidents, we established the potential safety accident causation structure of the project. The results are presented in Figure 3.

As illustrated in Figure 3, the potential safety accident causation structure of the project is divided into five hierarchical levels. From the bottom up, the first four levels represent the interrelation structure of identified safety risks, while the fifth level delineates various types of safety accidents. The first level, termed the environment safety risk level, encompasses most natural environment and management environment safety risks. The second level, named the personnel safety risk level, includes worker and manager safety risks. The third level, designated as the technology safety risk level, identifies technology-related safety risks. The fourth level focuses on machine safety risks, covering all machine-type safety risks. From a safety accident causality perspective, environment-type safety risks are considered root causes of potential safety accidents, whereas personnel-related and technology-type safety risks are fundamental causes. Machine-related safety risks serve as direct causes of potential safety accidents. Generally, most environment-type safety risks can influence personnel and technology-type safety risks, while personnel and technology-type safety risks often impact machine-type safety risks. Additionally, certain personnel-related safety risks, such as “safety management competency” and “safety inspection,” can also affect technology-type safety risks.

The hierarchical model presented in Figure 3 offers a structured approach to understanding how surface-level accidents, like “water inrush,” originate from latent root causes. Practitioners can utilize this model to enhance safety management and risk mitigation strategies in several ways. Firstly, prioritizing Level 1 factors, such as “insufficient geological surveys” and “high water pressure,” is crucial for preventing downstream failures. By addressing these foundational risks early, project managers can prevent more severe issues from arising later in the construction process. Secondly, mapping accident pathways based on the safety accident causation structure aids in designing cross-departmental emergency protocols. Understanding these sequences enables teams to coordinate with municipal utility providers for rapid pipeline repairs, thereby minimizing the impact of potential accidents. Pre-planning these responses ensures that all stakeholders are prepared and can respond swiftly to various scenarios. Thirdly, simplified versions of the causation structure serve as effective training tools for new engineers. Highlighting key risk factors and their correlations helps trainees recognize early warning signs. Regular training sessions improve workforce skills and foster a culture of proactive risk management and continuous learning.

## 6 Discussion and management implication

### 6.1 Discussion

The paper identified a new safety risk list for STUR shield construction based on a literature review and expert discussions. The list included 4 safety risk categories and 32 safety risks. The 4 safety risk categories include personnel-type safety risks, machine-type safety risks, technology-type safety risks, and environment-type safety risks. Personnel-type safety risks consist of worker safety risks and manager safety risks, while environment-type safety risks include natural environment safety risks and management environment safety risks. Firstly, compared to other research on STUR safety risk (28, 36, 48, 49), the safety risk list proposed in this study includes personnel-type and technology-type safety risks, as well as other safety risks (e.g., levee). Therefore, the safety risk list is more comprehensive and applicable across a wider range. Secondly, this taxonomy essentially follows the systems engineering paradigm of “personnel-machine-environment” (50). The key distinction is that we have separated technology-type safety risks from the broader category of machine-type safety risks in our list of safety risks. The reason is that construction technology is a more complex process or system involving the interaction rules among personnel, materials, and machine. Our classification is more concise and easier for practical applications. Moreover, most previous studies in shield construction safety management have also distinguished technology-related safety risks from other types of safety risks (21, 51, 52). Thirdly, compared to other safety risk identification frameworks [e.g., “personnel-machine-material-environment-management” (53), “personnel-machine-material-technology-environment” (23)] in construction safety management, we did not consider material-type safety risks. This decision was made because after multiple rounds of inspections, substandard materials should not be allowed into the construction process. Most material damage during construction occurs due to non-standard construction practices, and the associated safety risks can be categorized as personnel-type, machine-type, technology-type, and environment-type safety risks.

This research proposes a hybrid approach to analyze STUR shield construction safety risks and the interrelations among these risks by combining grey number theory, DEMATEL, and ISM. The approach was applied to a case to validate its feasibility. Firstly, little research has investigated the interrelation structure among STUR shield construction safety risks as highlighted previously. This research is the first to design an integrated method to analyze the interrelations of safety risks and establish a hierarchical structure for safety accident causation in this specific shielding context. Second, as mentioned earlier, several studies have explored the interrelations among safety risks in the extensive subway shield construction area using ISM, DEMATEL, or fuzzy ISM (29–32). One distinctive aspect of our approach is the incorporation of grey number theory into ISM and DEMATEL. Compared to research solely utilizing ISM and DEMATEL, the incorporation of grey numbers offers a method to address the uncertainty inherent in experts’ judgments. In addition, compared to studies using fuzzy ISM, our proposed approach can handle any distributed data, rely on fewer samples, and bypass the need to set different affiliation functions by utilizing grey number theory. Therefore, the method proposed in this paper has a broader range of applicability.

The proposed Grey-DEMATEL-ISM approach was applied to a case study involving the undercrossing of the Pearl River by Guangzhou Metro Line 18 from Pazhou West Station to Xiancun Station, to test its feasibility. The calculation processes and results demonstrated that this approach can effectively assess the significance of safety risks and determine their interrelation structure. Case validation showed that most natural environment safety risks (such as levees, quicksand layers, and high water pressure), management environment safety risks (including safety institutions and safety organization and duty), and managerial safety risks (like safety inspections and safety management competency) are prominent safety risks. These findings align with previous research in China. Firstly, natural environmental conditions have been identified as significant risks in subway engineering safety due to their complexity. For instance, Yue et al. (54) and Wang (55) investigated subway safety management during river crossings and highlighted that natural environment safety risks (such as geological and hydrological conditions) are primary factors leading to safety incidents like excavation face collapse, water inrush, and tunnel flooding. Additionally, Alagha and Chapman (56) and Zhu and Liang (57) emphasized that poor ground features (e.g., soft ground, proximity to piers) are particularly susceptible to causing safety accidents. Secondly, numerous previous studies have pointed out that management factors are also significant contributors to safety accidents, often due to inadequate safety management practices. For example, Zhang et al. (58) stressed that management-related issues are the most significant causes of safety accidents in China, noting that non-standard safety management practices remain prevalent at many construction sites. Xia et al. (59, 60) argued that safety managers play a pivotal role in safety management, and their characteristics and behavioral habits are primary causes of construction safety accidents. Therefore, the results of risk identification can provide valuable guidance for practical safety management practices.

Furthermore, the case study established a causation structure for potential safety accidents in shield tunneling undercrossing rivers (STUR) by assessing the interrelations among the identified safety risks using Interpretive Structural Modeling (ISM) calculations. The causation structure, from the bottom up, includes: environment safety risk level, personnel safety risk level, technology safety risk level, machine safety risk level, and accident level. This causation structure model essentially follows Heinrich’s Domino Theory (43), with one notable difference: the machine safety risk level is positioned at the direct cause level, whereas human-related safety risks are situated at a more distal level. This positioning is justified because a shield machine, as an integrated high-tech construction tool, provides a relatively enclosed and risk-isolated working environment for shield construction. Most personnel-related construction operations occur within the shield machine, making different types of machine-related failures the direct cause of most safety accidents. However, these machine failures may still be influenced by personnel-related factors. A similar concept is also found in the research by Chen et al. (5), who developed a causation model for subway shield construction. They argued that machine-related safety risks are more direct causes of shield construction safety accidents than human-related safety risks. Therefore, although this causation structure was established based on the case study, it can be widely applied to shield construction safety management.

### 6.2 Management implication

Based on the aforementioned analysis, several management policies can be proposed to enhance on-site safety STUR shield construction. Firstly, this research provides a comprehensive SCSR for STUR, which validates the “personnel-machine-technology-management” analytical framework. On-site safety managers can use this list to systematically identify and manage safety risks according to this framework. Secondly, the analysis highlights that management-related safety risks and managerial safety issues are significant. To address these, front-line managers can implement the following strategies: (a) develop safety institution procedures that are tailored to both China’s specific context and the unique requirements of the project; (b) establish a robust reward and punishment system within construction enterprises and among project managers, clearly defining their responsibilities and obligations, and enhancing supervision of the construction process; (c) enhance safety training programs to deepen managers’ understanding of safety management principles and improve their skills in implementing effective safety measures. Thirdly, natural environmental factors such as levees, quicksand layers, and high water pressure pose significant SCSRs of STUR. To mitigate these risks, project managers can take the following proactive measures: (a) develop and implement a reinforcement plan for levees if necessary before initiating the STUR shield construction; (b) conduct advance grouting to strengthen the soil layer and ensure its stability prior to the STUR shielding process; (c) control the excavation speed and continuously monitor water seepage in the tunnel to prevent potential incidents. These policies and countermeasures can help in effectively managing and reducing safety risks, thereby improving overall safety during STUR shield construction.

Moreover, the research findings can also inform policy-making efforts. Based on the evidence, we propose the following three policy recommendations. Firstly, government agencies should develop “Safety Management Guidelines for Shield Tunneling Under Rivers.” These guidelines should mandate pre-construction reinforcement plans for levees and grouting protocols for unstable strata. They should also require the implementation of dual prevention mechanisms that integrate our risk list for systematic risk identification and prioritization, alongside standardizing real-time monitoring of water pressure and grouting efficacy during construction. Secondly, to address pivotal antecedent risks such as “safety management competency” and “safety inspection,” national “Shield Construction Manager Certification Standards” should be established. These standards should include mandatory training modules focused on river-crossing geological challenges and emergency response strategies, require periodic competency assessments, and link promotion incentives to safety inspection performance metrics, such as hidden risk detection rates. This approach ensures that managers are well-prepared to handle the unique challenges associated with shield tunneling under rivers. Thirdly, given the predominance of technology and machine failures resulting from inadequate geological surveys and improper soil conditioning, “River-Crossing Shield Technology Approval Mechanisms” should be implemented. Such mechanisms should involve third-party verification of hydrogeological surveys using advanced technologies like LiDAR and borehole resistivity tomography. Additionally, pre-construction simulation testing of soil conditioning programs under high-water-pressure scenarios should be conducted, along with the use of blockchain-based documentation for grouting operations to ensure traceability and compliance. These measures will enhance the reliability and safety of shield tunneling projects crossing rivers.

## 7 Conclusion

The paper utilized literature review and expert discussions to identify safety risks during STUR shield construction. It integrated Grey number theory, DEMATEL, and ISM to propose a new approach for analyzing safety risks and their interrelations. A case study was selected to validate the feasibility of the approach. The research conclusions are as follows.

1 A more comprehensive SCSRs list of STUR is proposed, consisting of 4 safety risk categories: personnel-type safety risks, machine-type safety risks, technology-type safety risks, and environment-type safety risks, totaling 32 safety risks. Furthermore, the suggested safety risk list can be effectively utilized to identify potential safety risks associated with STUR shield construction.
2 A hybrid safety risk analysis approach was proposed by combining grey numbers, DEMATEL, and ISM. The feasibility of this approach in analyzing the significance and interrelation structure of identified safety risks was validated by applying it to a tunnel section of Guangzhou Metro Line 18.
3 The case study revealed that the most natural environment safety risks (levee, quicksand layer and high water pressure), management environment safety risks (safety institution and safety organization & duty) and manager safety risks (safety inspection, safety management competency) are salient safety risks. The accident causation structure, from the bottom up, includes environment safety risk level, personnel safety risk level, technology safety risk level, machine safety risk level, and accident level.
4 The research has two limitations. Firstly, we only selected one case to test the proposed approach in safety risk analysis. Therefore, future researchers can choose different types of cases to validate its broad applicability. Secondly, we solely examined the causality among the safety risks; subsequent researchers can analyze the coupling and loops of the safety risks.

## Data Availability

The original contributions presented in the study are included in the article/Supplementary material, further inquiries can be directed to the corresponding author/s.
