# Supplementary material for: Shield construction safety risks and their interrelations analysis of subway tunnel undercrossing a river based on Grey-DEMATEL-ISM
Source: Front Public Health. 2025 Apr 10;13:1536706. doi: 10.3389/fpubh.2025.1536706 (PMC12018409; doi:10.3389/fpubh.2025.1536706)
Supplement: Supplementary file 1 [file Data_Sheet_1.docx]

1. **Tables**

**Table 1. Shield construction safety risk list of STUR.**

| **Safety risk categories** | | **Safety risks** |
| --- | --- | --- |
| Personnel-type safety risks | Worker safety risks | W1: Physical and psychological health; W2: Safety awareness; W3: work experiences; W4: work skills |
|  | Manager safety risks | M1: Safety management intentions; M2: Safety management competency; M3: Safety communication; M4: Safety inspection. |
| Machine-type safety risks | | MA1: Cutter head system not working; MA2: Thrust cylinder system not working; MA3: Screw conveyor not working; MA4: Grouting system not working; MA5: Ventilation system not working; MA6: Electrical equipment not working. |
| Technology-type safety risks | | T1: Improper bank reinforcement technology; T2: Incomplete geological and hydrological survey; T3: Incomplete construction monitoring; T4: Improper excavation operation; T5: Improper soil conditioning program; T6: Improper grouting plan; T7: Improper sealed water-proof plan. |
| Environment-type safety risks | Natural environment safety risks | NE1: Levee; NE2: Shallow overburden layer; NE3: Quicksand layer; NE4: Exploration borehole; NE5: High water pressure; NE7: Underground voids. |
|  | Management environment safety risks | ME1: Safety culture; ME2: Safety institutions; ME3: Safety organization & duties; ME4: Safety training & education. |

**Table 2 The relationships between grey numbers and the evaluation degree.**

| **Evaluation expression** | **Evaluation degrees** | **Grey numbers** |
| --- | --- | --- |
| No impact | 0 | [0, 0] |
| Less impact | 1 | (0, 0.25] |
| Relative higher impact | 2 | (0.25, 0.5] |
| High impact | 3 | (0.5, 0.75] |
| Significant impact | 4 | (0.75, 1] |

**Table 3 The identified safety risks by the experts**

| **Safety risk categories** | | **Safety risks** |
| --- | --- | --- |
| Personnel-type safety risk | Worker safety risk | W2: Safety awareness; W3: Work experiences. |
|  | Manager safety risk | M2: Safety management competency; M4: Safety communication; M5: Safety inspection. |
| Machine-type safety risk | | MA3: Screw conveyor not working; MA4 Grouting system not working: MA5: Ventilation system not working; MA6: Electrical equipment not working. |
| Technology-type safety risk | | T2: Incomplete geological and hydrological investigation; T3: Incomplete construction monitoring; T4: Improper excavation operation; T5: Improper soil conditioning program; T6: Improper grouting program; T7: Improper sealed water-proof plan. |
| Environment-type safety risk | Natural environment safety risk | NE1: Levee; NE3: Quicksand layer; NE5: High water pressure. |
|  | Management environment safety risk | ME2: Safety institution; ME3: Safety organization & duty; ME4: Safety training & education. |

**Table 4 The direct impact matrix of safety risks the safety risks in Table 3.**

|  | W2 | W3 | M2 | M4 | M5 | MA3 | MA4 | MA5 | MA6 | T2 | T3 | T4 | T5 | T6 | T7 | NE2 | NE3 | NE5 | ME2 | ME3 | ME4 |
| --- | --- | --- | --- | --- | --- | --- | --- | --- | --- | --- | --- | --- | --- | --- | --- | --- | --- | --- | --- | --- | --- |
| W2 | 0.00 | 0.00 | 0.00 | 0.05 | 0.00 | 0.35 | 0.35 | 0.35 | 0.35 | 0.00 | 0.00 | 0.00 | 0.00 | 0.00 | 0.00 | 0.00 | 0.00 | 0.00 | 0.00 | 0.00 | 0.00 |
| W3 | 0.95 | 0.00 | 0.00 | 0.00 | 0.00 | 0.35 | 0.35 | 0.35 | 0.35 | 0.00 | 0.04 | 0.04 | 0.04 | 0.04 | 0.04 | 0.00 | 0.00 | 0.00 | 0.00 | 0.00 | 0.00 |
| M2 | 0.65 | 0.04 | 0.00 | 0.35 | 0.04 | 0.35 | 0.35 | 0.35 | 0.35 | 0.35 | 0.35 | 0.35 | 0.35 | 0.35 | 0.35 | 0.00 | 0.00 | 0.00 | 0.00 | 0.00 | 0.00 |
| M4 | 0.65 | 0.35 | 0.04 | 0.00 | 0.04 | 0.00 | 0.00 | 0.00 | 0.00 | 0.00 | 0.00 | 0.00 | 0.00 | 0.00 | 0.00 | 0.00 | 0.00 | 0.00 | 0.00 | 0.00 | 0.00 |
| M5 | 0.17 | 0.35 | 0.00 | 0.07 | 0.00 | 0.65 | 0.65 | 0.65 | 0.65 | 0.53 | 0.53 | 0.53 | 0.53 | 0.53 | 0.53 | 0.00 | 0.00 | 0.00 | 0.00 | 0.00 | 0.00 |
| MA3 | 0.00 | 0.00 | 0.00 | 0.00 | 0.00 | 0.00 | 0.00 | 0.00 | 0.01 | 0.00 | 0.00 | 0.00 | 0.00 | 0.00 | 0.00 | 0.00 | 0.00 | 0.00 | 0.00 | 0.00 | 0.00 |
| MA4 | 0.00 | 0.00 | 0.00 | 0.00 | 0.00 | 0.00 | 0.00 | 0.00 | 0.00 | 0.00 | 0.00 | 0.00 | 0.00 | 0.00 | 0.00 | 0.00 | 0.00 | 0.00 | 0.00 | 0.00 | 0.00 |
| MA5 | 0.00 | 0.00 | 0.00 | 0.00 | 0.00 | 0.00 | 0.00 | 0.00 | 0.00 | 0.00 | 0.00 | 0.00 | 0.00 | 0.00 | 0.00 | 0.00 | 0.00 | 0.00 | 0.00 | 0.00 | 0.00 |
| MA6 | 0.00 | 0.00 | 0.00 | 0.00 | 0.00 | 0.35 | 0.35 | 0.35 | 0.00 | 0.00 | 0.00 | 0.00 | 0.00 | 0.00 | 0.00 | 0.00 | 0.00 | 0.00 | 0.00 | 0.00 | 0.00 |
| T2 | 0.00 | 0.00 | 0.04 | 0.00 | 0.02 | 0.35 | 0.65 | 0.35 | 0.35 | 0.00 | 0.02 | 0.65 | 0.65 | 0.65 | 0.65 | 0.00 | 0.00 | 0.00 | 0.00 | 0.00 | 0.00 |
| T3 | 0.00 | 0.00 | 0.05 | 0.00 | 0.02 | 0.04 | 0.04 | 0.04 | 0.04 | 0.00 | 0.00 | 0.35 | 0.35 | 0.35 | 0.09 | 0.00 | 0.00 | 0.00 | 0.00 | 0.00 | 0.00 |
| T4 | 0.00 | 0.00 | 0.00 | 0.00 | 0.00 | 0.35 | 0.05 | 0.00 | 0.35 | 0.00 | 0.00 | 0.00 | 0.05 | 0.05 | 0.05 | 0.00 | 0.00 | 0.00 | 0.00 | 0.00 | 0.00 |
| T5 | 0.00 | 0.00 | 0.00 | 0.00 | 0.00 | 0.65 | 0.00 | 0.00 | 0.04 | 0.00 | 0.00 | 0.00 | 0.00 | 0.05 | 0.00 | 0.00 | 0.00 | 0.00 | 0.00 | 0.00 | 0.00 |
| T6 | 0.00 | 0.00 | 0.00 | 0.00 | 0.00 | 0.00 | 0.65 | 0.00 | 0.04 | 0.00 | 0.00 | 0.00 | 0.00 | 0.00 | 0.05 | 0.00 | 0.00 | 0.00 | 0.00 | 0.00 | 0.00 |
| T7 | 0.00 | 0.00 | 0.00 | 0.00 | 0.00 | 0.00 | 0.00 | 0.00 | 0.04 | 0.00 | 0.00 | 0.00 | 0.00 | 0.00 | 0.00 | 0.00 | 0.00 | 0.00 | 0.00 | 0.00 | 0.00 |
| NE1 | 0.00 | 0.00 | 0.00 | 0.35 | 0.35 | 0.35 | 0.03 | 0.04 | 0.04 | 0.41 | 0.04 | 0.41 | 0.41 | 0.41 | 0.41 | 0.00 | 0.00 | 0.00 | 0.00 | 0.00 | 0.00 |
| NE4 | 0.00 | 0.00 | 0.00 | 0.35 | 0.35 | 0.05 | 0.35 | 0.04 | 0.04 | 0.41 | 0.04 | 0.41 | 0.41 | 0.71 | 0.35 | 0.00 | 0.00 | 0.00 | 0.00 | 0.00 | 0.00 |
| NE5 | 0.00 | 0.00 | 0.00 | 0.35 | 0.35 | 0.00 | 0.35 | 0.00 | 0.04 | 0.07 | 0.04 | 0.41 | 0.07 | 0.65 | 0.71 | 0.00 | 0.00 | 0.00 | 0.00 | 0.00 | 0.00 |
| ME2 | 0.35 | 0.05 | 0.02 | 0.65 | 0.65 | 0.04 | 0.04 | 0.04 | 0.04 | 0.04 | 0.04 | 0.04 | 0.04 | 0.04 | 0.04 | 0.00 | 0.00 | 0.00 | 0.00 | 0.65 | 0.65 |
| ME3 | 0.41 | 0.04 | 0.03 | 0.65 | 0.71 | 0.01 | 0.01 | 0.04 | 0.04 | 0.04 | 0.04 | 0.04 | 0.04 | 0.04 | 0.04 | 0.00 | 0.00 | 0.00 | 0.00 | 0.00 | 0.65 |
| ME4 | 0.41 | 0.41 | 0.41 | 0.11 | 0.11 | 0.04 | 0.04 | 0.04 | 0.04 | 0.04 | 0.04 | 0.04 | 0.04 | 0.04 | 0.04 | 0.00 | 0.00 | 0.00 | 0.00 | 0.00 | 0.00 |

**Table 5 The comprehensive impact matrix of the safety risks in Table 3.**

|  | W2 | W3 | M2 | M4 | M5 | MA3 | MA4 | MA5 | MA6 | T2 | T3 | T4 | T5 | T6 | T7 | NE2 | NE3 | NE5 | ME2 | ME3 | ME4 |
| --- | --- | --- | --- | --- | --- | --- | --- | --- | --- | --- | --- | --- | --- | --- | --- | --- | --- | --- | --- | --- | --- |
| W2 | 0.00 | 0.00 | 0.00 | 0.03 | 0.00 | 0.23 | 0.23 | 0.23 | 0.22 | 0.00 | 0.00 | 0.00 | 0.00 | 0.00 | 0.00 | 0.00 | 0.00 | 0.00 | 0.00 | 0.00 | 0.00 |
| W3 | 0.60 | 0.00 | 0.00 | 0.00 | 0.00 | 0.27 | 0.27 | 0.27 | 0.26 | 0.00 | 0.03 | 0.03 | 0.03 | 0.03 | 0.03 | 0.00 | 0.00 | 0.00 | 0.00 | 0.00 | 0.00 |
| M2 | 0.44 | 0.04 | 0.00 | 0.22 | 0.03 | 0.32 | 0.32 | 0.28 | 0.28 | 0.22 | 0.22 | 0.26 | 0.26 | 0.26 | 0.25 | 0.00 | 0.00 | 0.00 | 0.00 | 0.00 | 0.00 |
| M4 | 0.44 | 0.22 | 0.03 | 0.01 | 0.03 | 0.04 | 0.04 | 0.04 | 0.04 | 0.00 | 0.00 | 0.01 | 0.01 | 0.01 | 0.01 | 0.00 | 0.00 | 0.00 | 0.00 | 0.00 | 0.00 |
| M5 | 0.15 | 0.22 | 0.01 | 0.05 | 0.00 | 0.54 | 0.53 | 0.48 | 0.48 | 0.33 | 0.34 | 0.39 | 0.39 | 0.39 | 0.38 | 0.00 | 0.00 | 0.00 | 0.00 | 0.00 | 0.00 |
| MA3 | 0.00 | 0.00 | 0.00 | 0.00 | 0.00 | 0.00 | 0.00 | 0.00 | 0.01 | 0.00 | 0.00 | 0.00 | 0.00 | 0.00 | 0.00 | 0.00 | 0.00 | 0.00 | 0.00 | 0.00 | 0.00 |
| MA4 | 0.00 | 0.00 | 0.00 | 0.00 | 0.00 | 0.00 | 0.00 | 0.00 | 0.00 | 0.00 | 0.00 | 0.00 | 0.00 | 0.00 | 0.00 | 0.00 | 0.00 | 0.00 | 0.00 | 0.00 | 0.00 |
| MA5 | 0.00 | 0.00 | 0.00 | 0.00 | 0.00 | 0.00 | 0.00 | 0.00 | 0.00 | 0.00 | 0.00 | 0.00 | 0.00 | 0.00 | 0.00 | 0.00 | 0.00 | 0.00 | 0.00 | 0.00 | 0.00 |
| MA6 | 0.00 | 0.00 | 0.00 | 0.00 | 0.00 | 0.22 | 0.22 | 0.22 | 0.00 | 0.00 | 0.00 | 0.00 | 0.00 | 0.00 | 0.00 | 0.00 | 0.00 | 0.00 | 0.00 | 0.00 | 0.00 |
| T2 | 0.00 | 0.00 | 0.03 | 0.00 | 0.01 | 0.30 | 0.47 | 0.24 | 0.25 | 0.00 | 0.02 | 0.41 | 0.41 | 0.42 | 0.42 | 0.00 | 0.00 | 0.00 | 0.00 | 0.00 | 0.00 |
| T3 | 0.00 | 0.00 | 0.03 | 0.00 | 0.01 | 0.07 | 0.06 | 0.03 | 0.04 | 0.00 | 0.00 | 0.22 | 0.22 | 0.23 | 0.06 | 0.00 | 0.00 | 0.00 | 0.00 | 0.00 | 0.00 |
| T4 | 0.00 | 0.00 | 0.00 | 0.00 | 0.00 | 0.24 | 0.05 | 0.01 | 0.22 | 0.00 | 0.00 | 0.00 | 0.03 | 0.03 | 0.03 | 0.00 | 0.00 | 0.00 | 0.00 | 0.00 | 0.00 |
| T5 | 0.00 | 0.00 | 0.00 | 0.00 | 0.00 | 0.41 | 0.00 | 0.00 | 0.03 | 0.00 | 0.00 | 0.00 | 0.00 | 0.03 | 0.00 | 0.00 | 0.00 | 0.00 | 0.00 | 0.00 | 0.00 |
| T6 | 0.00 | 0.00 | 0.00 | 0.00 | 0.00 | 0.00 | 0.41 | 0.00 | 0.03 | 0.00 | 0.00 | 0.00 | 0.00 | 0.00 | 0.03 | 0.00 | 0.00 | 0.00 | 0.00 | 0.00 | 0.00 |
| T7 | 0.00 | 0.00 | 0.00 | 0.00 | 0.00 | 0.00 | 0.00 | 0.00 | 0.03 | 0.00 | 0.00 | 0.00 | 0.00 | 0.00 | 0.00 | 0.00 | 0.00 | 0.00 | 0.00 | 0.00 | 0.00 |
| NE1 | 0.03 | 0.02 | 0.00 | 0.22 | 0.22 | 0.31 | 0.11 | 0.07 | 0.09 | 0.28 | 0.04 | 0.31 | 0.31 | 0.31 | 0.31 | 0.00 | 0.00 | 0.00 | 0.00 | 0.00 | 0.00 |
| NE4 | 0.03 | 0.02 | 0.00 | 0.22 | 0.22 | 0.13 | 0.33 | 0.07 | 0.09 | 0.28 | 0.04 | 0.31 | 0.31 | 0.50 | 0.27 | 0.00 | 0.00 | 0.00 | 0.00 | 0.00 | 0.00 |
| NE5 | 0.03 | 0.02 | 0.00 | 0.22 | 0.22 | 0.06 | 0.30 | 0.03 | 0.08 | 0.06 | 0.04 | 0.28 | 0.07 | 0.44 | 0.48 | 0.00 | 0.00 | 0.00 | 0.00 | 0.00 | 0.00 |
| ME2 | 0.36 | 0.11 | 0.05 | 0.47 | 0.47 | 0.13 | 0.13 | 0.12 | 0.12 | 0.07 | 0.07 | 0.08 | 0.08 | 0.09 | 0.08 | 0.00 | 0.00 | 0.00 | 0.00 | 0.41 | 0.45 |
| ME3 | 0.36 | 0.10 | 0.05 | 0.43 | 0.46 | 0.11 | 0.11 | 0.11 | 0.11 | 0.07 | 0.07 | 0.08 | 0.08 | 0.08 | 0.08 | 0.00 | 0.00 | 0.00 | 0.00 | 0.00 | 0.41 |
| ME4 | 0.33 | 0.27 | 0.26 | 0.09 | 0.07 | 0.10 | 0.10 | 0.09 | 0.09 | 0.05 | 0.05 | 0.05 | 0.05 | 0.06 | 0.05 | 0.00 | 0.00 | 0.00 | 0.00 | 0.00 | 0.00 |

**Table 6 The center degree and cause degree of the safety risks in Table 3.**

| **Number** | **Safety risks** | **D** | **C** | **CD** | **RBCD** | **RD** | **RBRD** |
| --- | --- | --- | --- | --- | --- | --- | --- |
| 1 | W2: Safety awareness | 0.95 | 2.78 | 3.74 | 4 | -1.8 | 13 |
| 2 | W3: Work experiences | 1.8 | 1.04 | 2.84 | 13 | 0.75 | 10 |
| 3 | M2: Safety management competency | 3.41 | 0.46 | 3.86 | 3 | 2.95 | 2 |
| 4 | M4: Safety communication | 0.92 | 1.96 | 2.89 | 12 | -1 | 12 |
| 5 | M5: Safety inspection | 4.67 | 1.74 | 6.41 | 1 | 2.92 | 3 |
| 6 | MA3: Screw conveyor not working | 0.01 | 3.47 | 3.47 | 6 | -3.5 | 20 |
| 7 | MA4: Grouting system not working | 0 | 3.69 | 3.69 | 5 | -3.7 | 21 |
| 8 | MA5: Ventilation system not working | 0 | 2.29 | 2.29 | 20 | -2.3 | 17 |
| 9 | MA6: Electrical equipment not working | 0.66 | 2.45 | 3.11 | 9 | -1.8 | 14 |
| 10 | T2: Incomplete geological and hydrological investigation | 2.99 | 1.36 | 4.35 | 2 | 1.62 | 8 |
| 11 | T3: Incomplete construction monitoring | 0.99 | 0.93 | 1.92 | 21 | 0.06 | 11 |
| 12 | T4: Improper excavation operation | 0.61 | 2.43 | 3.04 | 11 | -1.8 | 15 |
| 13 | T5: Improper soil conditioning program | 0.47 | 2.27 | 2.74 | 15 | -1.8 | 16 |
| 14 | T6: Improper grouting program | 0.47 | 2.87 | 3.34 | 7 | -2.4 | 18 |
| 15 | T7: Improper sealed water-proof plan | 0.03 | 2.48 | 2.51 | 18 | -2.5 | 19 |
| 16 | NE1: Levee | 2.65 | 0 | 2.65 | 16 | 2.65 | 5 |
| 17 | NE3: Quicksand layer | 2.84 | 0 | 2.84 | 14 | 2.84 | 4 |
| 18 | NE5: High water pressure | 2.35 | 0 | 2.35 | 19 | 2.35 | 6 |
| 19 | ME2: Safety institution | 3.28 | 0 | 3.28 | 8 | 3.28 | 1 |
| 20 | ME3: Safety organization & duty | 2.69 | 0.41 | 3.1 | 10 | 2.28 | 7 |
| 21 | ME4: Safety training & education | 1.7 | 0.9 | 2.6 | 17 | 0.8 | 9 |

Notes: CD refers to center degree; RD refers to cause degree; RBCD means ranked by DC; RBRD means ranked by RD.

**Table 7 The accessible matrix of the safety risks in Table 3.**

|  | W2 | W3 | M2 | M4 | M5 | MA3 | MA4 | MA5 | MA6 | T2 | T3 | T4 | T5 | T6 | T7 | NE2 | NE3 | NE5 | ME2 | ME3 | ME4 |
| --- | --- | --- | --- | --- | --- | --- | --- | --- | --- | --- | --- | --- | --- | --- | --- | --- | --- | --- | --- | --- | --- |
| W2 | 0 | 0 | 0 | 0 | 0 | 1 | 1 | 1 | 1 | 0 | 0 | 0 | 0 | 0 | 0 | 0 | 0 | 0 | 0 | 0 | 0 |
| W3 | 1 | 0 | 0 | 0 | 0 | 1 | 1 | 1 | 1 | 0 | 0 | 0 | 0 | 0 | 0 | 0 | 0 | 0 | 0 | 0 | 0 |
| M2 | 1 | 0 | 0 | 1 | 0 | 1 | 1 | 1 | 1 | 1 | 1 | 1 | 1 | 1 | 1 | 0 | 0 | 0 | 0 | 0 | 0 |
| M4 | 1 | 1 | 0 | 0 | 0 | 0 | 0 | 0 | 0 | 0 | 0 | 0 | 0 | 0 | 0 | 0 | 0 | 0 | 0 | 0 | 0 |
| M5 | 0 | 1 | 0 | 0 | 0 | 1 | 1 | 1 | 1 | 1 | 1 | 1 | 1 | 1 | 1 | 0 | 0 | 0 | 0 | 0 | 0 |
| MA3 | 0 | 0 | 0 | 0 | 0 | 0 | 0 | 0 | 0 | 0 | 0 | 0 | 0 | 0 | 0 | 0 | 0 | 0 | 0 | 0 | 0 |
| MA4 | 0 | 0 | 0 | 0 | 0 | 0 | 0 | 0 | 0 | 0 | 0 | 0 | 0 | 0 | 0 | 0 | 0 | 0 | 0 | 0 | 0 |
| MA5 | 0 | 0 | 0 | 0 | 0 | 0 | 0 | 0 | 0 | 0 | 0 | 0 | 0 | 0 | 0 | 0 | 0 | 0 | 0 | 0 | 0 |
| MA6 | 0 | 0 | 0 | 0 | 0 | 1 | 1 | 1 | 0 | 0 | 0 | 0 | 0 | 0 | 0 | 0 | 0 | 0 | 0 | 0 | 0 |
| T2 | 0 | 0 | 0 | 0 | 0 | 1 | 1 | 1 | 1 | 0 | 0 | 1 | 1 | 1 | 1 | 0 | 0 | 0 | 0 | 0 | 0 |
| T3 | 0 | 0 | 0 | 0 | 0 | 0 | 0 | 0 | 0 | 0 | 0 | 1 | 1 | 1 | 0 | 0 | 0 | 0 | 0 | 0 | 0 |
| T4 | 0 | 0 | 0 | 0 | 0 | 1 | 0 | 0 | 1 | 0 | 0 | 0 | 0 | 0 | 0 | 0 | 0 | 0 | 0 | 0 | 0 |
| T5 | 0 | 0 | 0 | 0 | 0 | 1 | 0 | 0 | 0 | 0 | 0 | 0 | 0 | 0 | 0 | 0 | 0 | 0 | 0 | 0 | 0 |
| T6 | 0 | 0 | 0 | 0 | 0 | 0 | 1 | 0 | 0 | 0 | 0 | 0 | 0 | 0 | 0 | 0 | 0 | 0 | 0 | 0 | 0 |
| T7 | 0 | 0 | 0 | 0 | 0 | 0 | 0 | 0 | 0 | 0 | 0 | 0 | 0 | 0 | 0 | 0 | 0 | 0 | 0 | 0 | 0 |
| NE1 | 0 | 0 | 0 | 1 | 1 | 1 | 0 | 0 | 0 | 1 | 0 | 1 | 1 | 1 | 1 | 0 | 0 | 0 | 0 | 0 | 0 |
| NE4 | 0 | 0 | 0 | 1 | 1 | 0 | 1 | 0 | 0 | 1 | 0 | 1 | 1 | 1 | 1 | 0 | 0 | 0 | 0 | 0 | 0 |
| NE5 | 0 | 0 | 0 | 1 | 1 | 0 | 1 | 0 | 0 | 0 | 0 | 1 | 0 | 1 | 1 | 0 | 0 | 0 | 0 | 0 | 0 |
| ME2 | 1 | 0 | 0 | 1 | 1 | 0 | 0 | 0 | 0 | 0 | 0 | 0 | 0 | 0 | 0 | 0 | 0 | 0 | 0 | 1 | 1 |
| ME3 | 1 | 0 | 0 | 1 | 1 | 0 | 0 | 0 | 0 | 0 | 0 | 0 | 0 | 0 | 0 | 0 | 0 | 0 | 0 | 0 | 1 |
| ME4 | 1 | 1 | 1 | 0 | 0 | 0 | 0 | 0 | 0 | 0 | 0 | 0 | 0 | 0 | 0 | 0 | 0 | 0 | 0 | 0 | 0 |

1. **Equations**

${\underline{\bigoplus}\tilde{e}}_{ij}^{k}=({\underline{\oplus}e}_{ij}^{k}-min{\underline{\oplus}e}_{ij}^{k})/(max\overline{\oplus}e_{ij}^{k}-min{\underline{\bigoplus}e}_{ij}^{k})$ (1)

$\overline{\oplus}\tilde{e}_{ij}^{k}=({\overline{\oplus}e}_{ij}^{k}-min{\overline{\oplus}e}_{ij}^{k})/(max\overline{\oplus}e_{ij}^{k}-min{\underline{\bigoplus}e}_{ij}^{k})$ (2)

$y_{ij}^{k}=[{\underline{\bigoplus}\tilde{e}}_{ij}^{k}(1-{\underline{\bigoplus}\tilde{e}}_{ij}^{k})+(\overline{\oplus}\tilde{e}_{ij}^{k}\cdot{\overline{\oplus}e}_{ij}^{k})]/(1-{\underline{\bigoplus}\tilde{e}}_{ij}^{k}+\overline{\oplus}\tilde{e}_{ij}^{k})$ (3)

$\bar{e}_{ij}^{k}=min{\underline{\oplus}e}_{ij}^{k}+y_{ij}^{k}(max\overline{\oplus}e_{ij}^{k}-min{\underline{\bigoplus}e}_{ij}^{k})$ (4)

$E^{'}=\left[ \begin{matrix} 0 & e_{12}^{'} & \ldots& e_{1n}^{'} \\ e_{21}^{'} & 0 & \ldots& e_{2n}^{'} \\ \vdots& \vdots& \ldots& \vdots\\ e_{n1}^{'} & e_{n2}^{'} & \ldots& 0 \end{matrix} \right]={(e_{ij}^{'})}_{n\times n}$ (5)

$B=\frac{e_{ij}^{'}}{\text{max}(\sum_{j=1}^{n} e_{ij}^{'})}=\left( b_{\text{ij}} \right)_{n\times n} (i,j=1, 2, 3,\cdots n)$ (6)

$T=B+B_{1}+B_{2}+B_{3}+\cdots+B_{n}=B\left( I-B \right)^{-1}={(t_{ij})}_{n\times n}$ (7)

${CD}_{i}=D_{i}+F_{i}，(i=1, 2, 3,\cdots n)$ (8)

${RD}_{i}=D_{i}-F_{i}，(i=1, 2, 3,\cdots n)$ (9)

$D_{i}=\sum_{j=1}^{n} t_{ij}，(i=1, 2, 3, \cdots n)$ (10)

$C_{i}=\sum_{i=1}^{n} t_{ij}，(j=1, 2, 3, \cdots n)$ (11)

$H=T+I$ (12)

$\left\{ \begin{matrix} r_{ij}=1，h_{ij}\geq\lambda(i,j=1,2,\cdots,n) \\ r_{ij}=0，h_{ij}\leq\lambda(i,j=1,2,\cdots,n) \end{matrix} \right.$ (13)
